# Cretaceous to early Paleogene sediment provenance transition from continental to magmatic arc systems in the Northwestern Pacific Region

**DOI:** 10.1038/s41598-024-55471-1

**Published:** 2024-03-27

**Authors:** Harisma Harisma, Sota Niki, Takafumi Hirata, Hajime Naruse

**Affiliations:** 1 Department of Geological Engineering, Halu Oleo University, Kampus Hijau Bumi Tridharma, Anduonohu, Kendari, Sulawesi Tenggara 93232 Indonesia; 2 Geochemical Research Center, The University of Tokyo, Hongo, Bunkyo-ku, Tokyo, 113-0033 Japan; 3 Department of Geology and Mineralogy, Kyoto University, Kitashirakawa Oiwake-cho, Sakyo-ku, Kyoto, 606-8502 Japan

**Keywords:** Sedimentology, Tectonics

## Abstract

Unraveling the Paleo-Kuril Arc's origins is key to understanding northwest Pacific tectonics. The Paleo-Kuril Arc is viewed as an intraoceanic arc from trench subduction between the Izanagi and Pacific Plates. Alternatively, several studies suggest the Paleo-Kuril Arc as a continental magmatic arc, hypothesizing the existence of a mid-oceanic ridge and Paleogene subduction, placing the Paleo-Kuril Arc near the Okhotsk Block's southern edge. This study clarifies these hypotheses, previously clouded by limited geochronological data on deposits in the Paleo-Kuril Arc. We conducted U–Pb dating to examine the origins of detrital zircons from the Cretaceous–Paleogene Tokoro and Nemuro Belts of the Paleo-Kuril Arc. Cluster analysis, merging new and existing data, identified two unique detrital zircon age clusters. The abundance of Precambrian zircons in Cretaceous–Paleocene Paleo-Kuril Arc sandstones (Type 1 Cluster) suggests a continental magmatic origin, supporting the ridge subduction model. However, an early Eocene shift to a consistent local volcanic source (Type 2 Cluster) highlights a significant provenance change. This geochronological evidence, indicating a separation from continental sources, calls for further research to decode the simultaneous development of sediment sources in different geological belts, potentially tied to the ridge subduction event.

## Introduction

The Izanagi Plate was an ancient oceanic plate in the northwest (NW) Pacific region predicted by the age distribution of the oceanic crust of the Pacific Plate^1^. There must have been a mid-oceanic ridge between these two oceanic plates. It has been suggested that the Izanagi Plate vanished when this ridge subducted into the continental plates in the North Pacific region^2–4^. Many studies considered such ridge subduction as one of the most significant tectonic events in this region^1–3^, which may have caused the cessation of volcanism and uplifting around the subduction zone.

**Figure 1:**
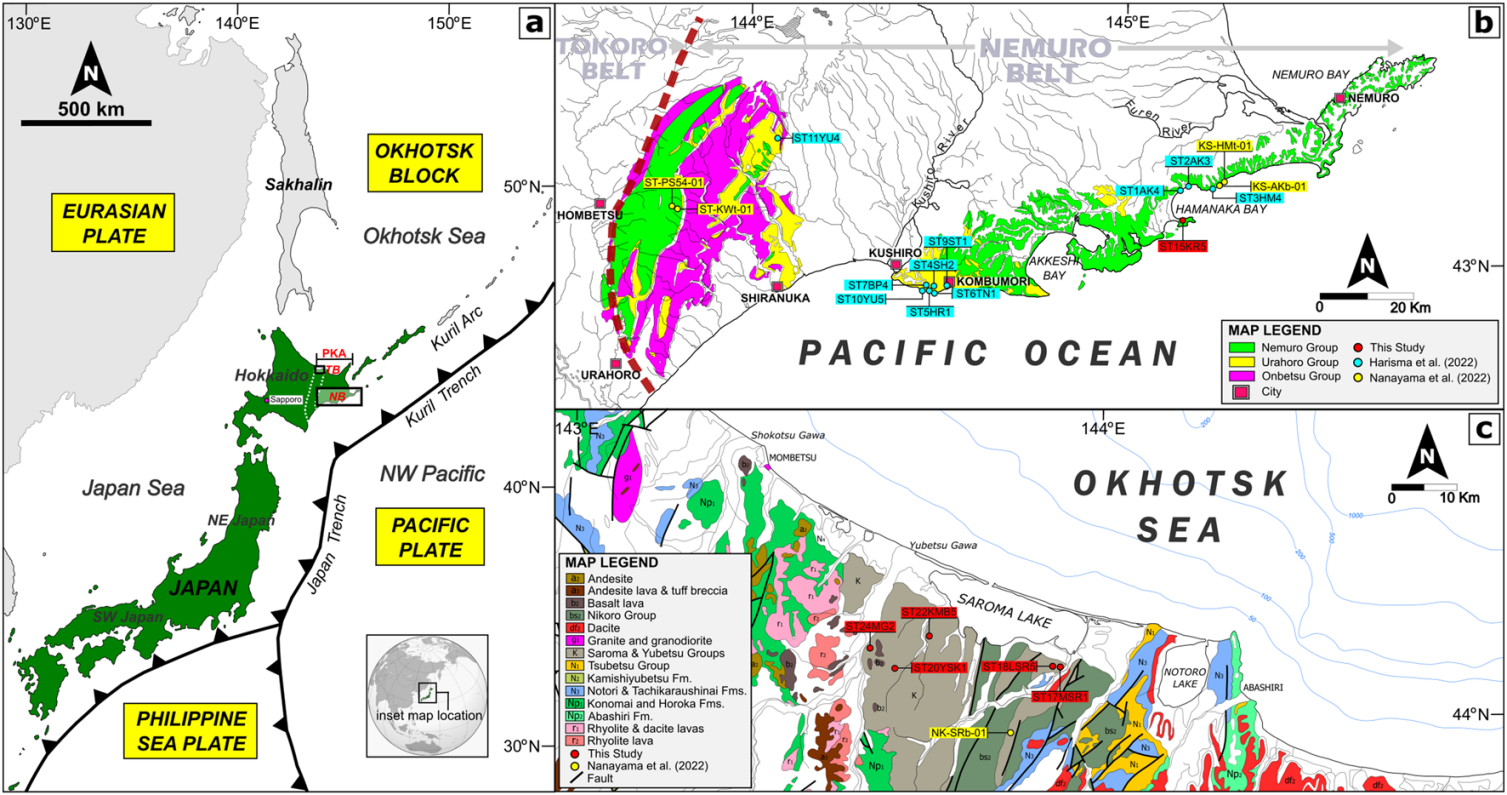
Geological map of the study area. (**a**) Location of Japan Island in the NW Pacific region. The globe figure (below figure) was obtained from https://en.wikipedia.org/wiki/File:Japan_(orthographic_projection).svg. *NB* Nemuro Belt, *PKA* Paleo-Kuril Arc, *TB* Tokoro Belt. (**b**) Geologic map of the Nemuro Belt and sample localities (modified after Harisma et al.^6^). (**c**) Geologic map of the Tokoro Belt and sample localities (modified from GeomapNavi, 2023; https://gbank.gsj.jp/geonavi/datastore/).

However, a recent study^2^ suggested that the boundary between the Izanagi and Pacific Plates was the subduction zone and, thus, that ridge subduction did not occur in the Paleogene. One of their primary pieces of evidence is that the Paleo-Kuril Arc system in Hokkaido Island in northern Japan (Fig. 1a) originated as an intraoceanic arc system, which was indicative of the existence of a subduction zone between two plates^2^. Meanwhile, paleomagnetic studies suggested that the Paleo-Kuril Arc could have developed in low-latitude areas. Thus, Bazhenov et al.^5^ interpreted the Paleo-Kuril Arc as an intraoceanic magmatic arc system between the Izanagi and Pacific Plates.

The recent geochronological analysis of Harisma et al.^6^ in the Late Cretaceous–Paleogene Nemuro Belt implied that the sediment of the Paleo-Kuril Arc was not only supplied with sediments from rocks of its local igneous origin but also from the Precambrian continental crust, suggesting that the Paleo-Kuril Arc originated along the southern end of the Okhotsk Block as a continental arc system during the Late Cretaceous. In contrast, another geochronological study suggested that the Paleo-Kuril Arc could have developed as an immature oceanic island arc system in the Late Cretaceous^7^ because it did not detect detrital zircon grains before 85 Ma in the basal successions of the Tokoro Belt, which is part of the Paleo-Kuril Arc system^7^. The geochemical analysis of the igneous rocks in the Nemuro Belt also suggested that the Paleo-Kuril Arc may have developed as an immature magmatic arc system^8^. Thus, the discussion on the origin of the arc has yet to be settled.

Contradictory interpretations of the origin of the Paleo-Kuril Arc have been proposed probably because the areas interpreted as the Paleo-Kuril Arc may have not been a single tectonic terrain initially. For instance, the Nemuro and Tokoro Belts (Fig. 1b,c) had been interpreted as tectonic belts that belonged to the Paleo-Kuril Arc system^2,5,9^. However, Bazhenov et al.^5^ argued that these two tectonic belts belonged to different intraoceanic island arc systems and that they were accreted to the NE Japan Arc system at different timings. This hypothesis can be verified by examining whether the Tokoro Belt recorded a sediment provenance transition similar to that in the Nemuro Belt, indicated by the detrital zircon U–Pb age distributions. Harisma et al.^6^ reported that the sediment provenance of the Nemuro Belt changed from multiple-source (continental crusts and magmatic arcs) to single-source (local magmatic arc) regions. If the Tokoro Belt was part of the Paleo-Kuril Arc, the provenance transition observed in the Nemuro Belt should have also been recorded in this tectonic belt.

Aiming to conclude the discussions on the origin of the Paleo-Kuril Arc, we report the U–Pb age distributions of the detrital zircon grains newly obtained from sandstone samples of the Late Cretaceous Saroma and the Late Cretaceous–Paleogene Yubetsu Groups in the Tokoro Belt distributed in eastern Hokkaido. We then conducted statistical analyses with integrated datasets using existing detrital zircon data from the Nemuro and Tokoro Belts^6,7,10^ to provide new insights into the link between the two tectonic belts and the evolution of the Paleo-Kuril Arc.

## Tectonic evolution of the Paleo-Kuril Arc

The Paleo-Kuril arc-trench system is the system that developed along a past plate subduction zone. The Kuril Arc is now connected to the Northeast Japan Arc at the mid-axis of Hokkaido Island. When the two trench systems conjunct each other, significant deformation of the island arc occurred, and tectonic belt rearrangement occurred. In this study, we distinguish the system before such large-scale deformation from the modern Kuril Arc-Trench system and define it as the Paleo-Kuril Arc-Trench system^11^.

The Paleo-Kuril Arc system is exposed in the eastern part of Hokkaido and consists of two tectonic belts: the Nemuro and Tokoro Belts (Fig. 1a). The Nemuro Belt comprises of three groups (the Upper Cretaceous to Paleogene Nemuro Group, the Eocene Urahoro Group, and the Oligocene Onbetsu Group), and unconformities are existing between these groups. The Nemuro Group has been interpreted to be forearc basin deposits. This group is the Upper Cretaceous to Paleogene succession, consisting of marine clastic deposits and has been divided into nine formations (the Nokkamappu, Otadai, Monshizu, Oborogawa, Hamanaka, Akkeshi, Tokotan, Kiritappu, and Shiomi Formations, from older to younger). The Urahoro Group is the Eocene succession, consisting mainly of fluvial and brackish deposits. This group is divided into six formations (the Beppo, Harutori, Tenneru, Yuubetsu, Shitakara, and Shakubetsu Formations, from older to younger).

The Tokoro Belt is another tectonic belt of the Paleo-Kuril Arc composed of forearc basin deposits and the accretionary complexes (the Nikoro, Saroma, and Yubetsu Groups). This belt is located in the eastern Hokkaido (Fig. 1a) and extends from north to south, exceeding 150 km long and up to 40 km wide. The Saroma Group is the Late Cretaceous deposits, consisting of sedimentary sequences of conglomerate, sandstone, and mudstone. It unconformably overlies the Nikoro Group^12,13^. This group is divided into three units (the Lower, Middle, and Upper) based on their lithology and has been interpreted as forearc basin deposits^14^. The Yubutsu Group is the Late Cretaceous to Paleogene successions and has been considered as accretionary complex deposits. This group is subdivided into ten units: the Kumanosawa, Mukaiengaru, Toyosato, Yasukuni, Asahino, Kamibaro, Onari, Mizuho, Wakasa, and Nakazono Units in ascending stratigraphic order. A previous study suggested that the boundary between these lithologic units are faults^15^. The reverse fault separates the Yubetsu Group from the Nikoro and Saroma Groups^15,16^.

The tectonic evolution of the Paleo-Kuril Arc before the Miocene can be summarized as follows according to geophysical and geological studies. The Paleo-Kuril Arc developed as a magmatic arc in a subduction zone in the Late Cretaceous. An accretionary complex (the Nikoro Group of the Tokoro Belt) and forearc basin deposits (the Saroma and Nemuro Groups) were formed along the subduction margin. However, as described earlier, the locations of the subducting plate and the subduction zone are yet controversial^2,3,6^. The unconformity between the Nemuro and Urahoro Groups also developed from the Paleocene to the Early Eocene (62–54 Ma)^3^. The unconformity between the Nemuro and Urahoro Groups has been attributed to a ridge subduction of the Izanagi-Pacific Plates. Kimura et al.^3^ found that the unconformity gap formed in the forearc basin along the northern Pacific region that occurred non-simultaneously, starting from SW Japan, NE Japan, central Hokkaido, and later in east Hokkaido. They suggested that the period of unconformity is consistent with the Izanagi-Pacific ridge subduction. The interpretation of Kimura et al.^3^ is conformably with the plate configuration reconstruction that was proposed by Seton et al.^1^. Moreover, study from Wu and Wu^17^ also identified a gap of magmatic activity that occurred during early Eocene (56–46 Ma) along the continental margin in the NE Asian region, which attributed to the ridge subduction event. Thus, it is reasonable to interpret that the unconformity between the Nemuro and Urahoro Groups is related to the ridge subduction of the Izanagi-Pacific Plates. On the other hand, if the ridge subduction did not occur in the North Pacific during the Paleogene as Domeier et al.^2^ and Bazhenov et al.^5^, and others have argued^1,3,6^, then a different model must be considered for the cause of the simultaneous development of these unconformities.

In the Middle Eocene to the Oligocene, a clockwise rotation of more than 70° occurred in the western part of the Paleo-Kuril Arc, implying the tectonic bending of the magmatic arc^18,19^. Katagiri et al.^19^ suggested that the oblique collision of the Paleo-Kuril Arc to eastern margin of the Eurasian Plate caused this bending of the tectonic belts^20–22^.

## Results

### U–Pb age distributions of detrital zircon grains in the Paleo-Kuril Arc

**Figure 2:**
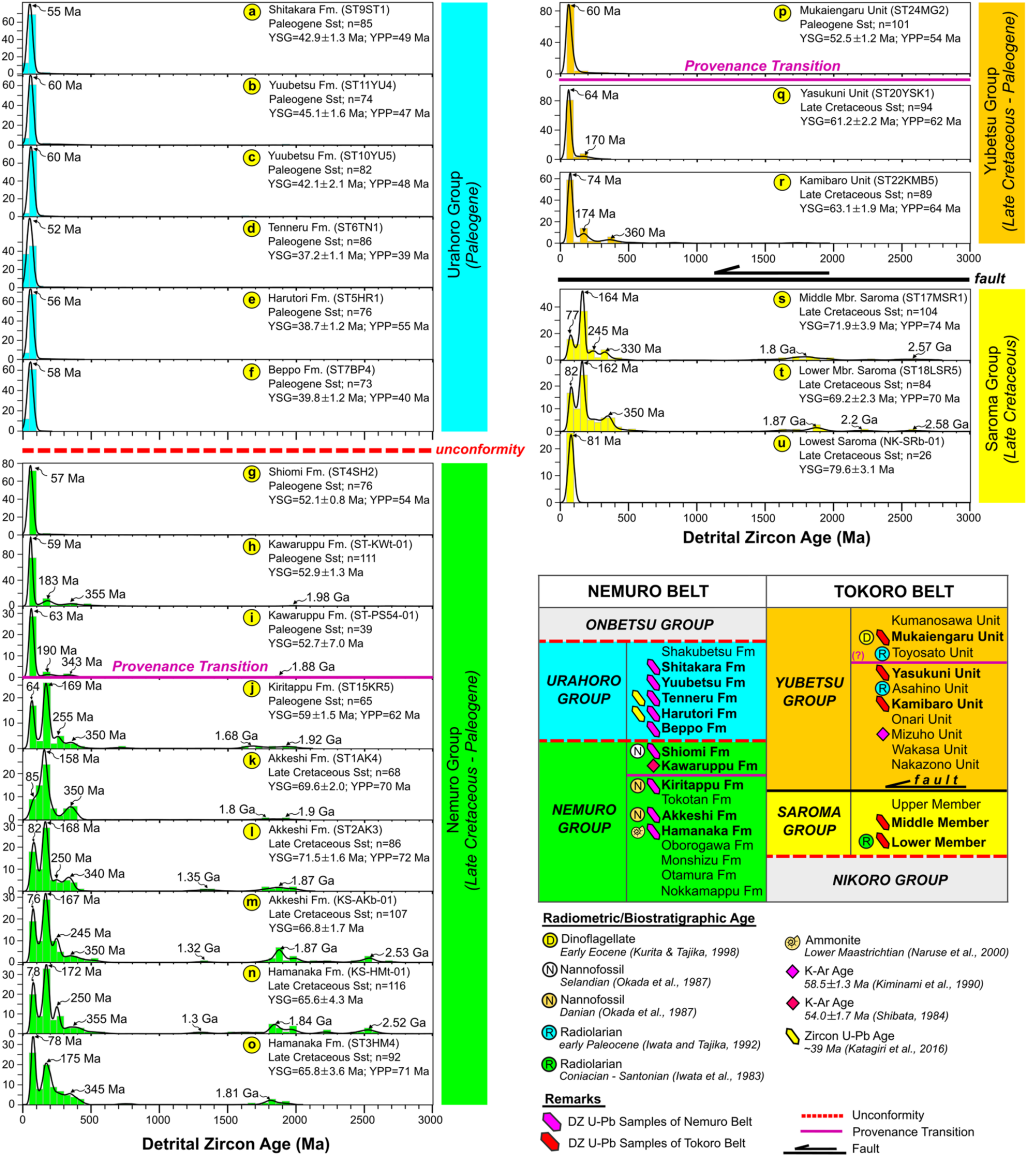
Detrital zircon U–Pb age distributions from samples of the Nemuro and Tokoro Belts. Blackline: KDE has a 20-Myr bandwidth, and histograms have a 50-Myr bandwidth. The stratigraphic column of the Nemuro Belt based on Kiminami^23^, Matsui^24^, and Okada et al.^25^, while the Tokoro Belt based on Iwata and Tajika^26^. In the Nemuro Belt, radiometric and biostratigraphic ages were analyzed, including zircon U–Pb ages^19,27^, and K–Ar dating^28^, nannofossil^25^, and ammonite^29^. While in the Tokoro Belt, radiometric and biostratigraphic ages were analyzed, such as: Dinoflagellate^30^, Radiolarians^31,32^, and K–Ar dating^33^. Detrital zircon U–Pb analysis from Harisma et al.^6^ (**a**–**g**, **k**,**l**,**o**); Nanayama et al.^7^ (**h**,**i**,**m**,**n**,**u**). *n* number of zircon grain analyses, *Fm* formation, *Mbr* member.

We examined six samples of sedimentary rocks in the Paleo-Kuril Arc, consisting of five sandstone samples from the Saroma and Yubetsu Groups and one sandstone sample from the Nemuro Group (Fig. 2). In these samples, a total of 537 grains were analyzed, and the U–Pb age distributions of the detrital zircon grains ranged from the Archean to the Eocene. The samples from the middle (ST17MSR1) and lower (ST18LSR5) members of the Saroma Group and the Kiritappu Formation (ST15KR5) of the Nemuro Group were characterized by age peaks of the Phanerozoic (72–77, 162–164, and 330 Ma) and the Precambrian (1.8 and 2.6 Ga). The prominent peak was Jurassic (162–164 Ma), and the others were subordinate peaks. In contrast, the samples from the Yubetsu Group lacked age peaks from the Precambrian. They exhibited U–Pb age distributions of detrital zircon grains with peaks only in the Phanerozoic (61–74, 170, and 360 Ma). Jurassic (170 Ma) and Paleozoic (360 Ma) peaks were weak in these samples, and the Paleozoic peak could not be detected in the sample of the Yasukuni Formation. The sample from the Mukaiengaru Formation exhibited a unimodal peak at the Paleocene (61 Ma) (Fig. 2).

### Analysis of Bayesian population correlations and classification of age distributions

**Figure 3:**
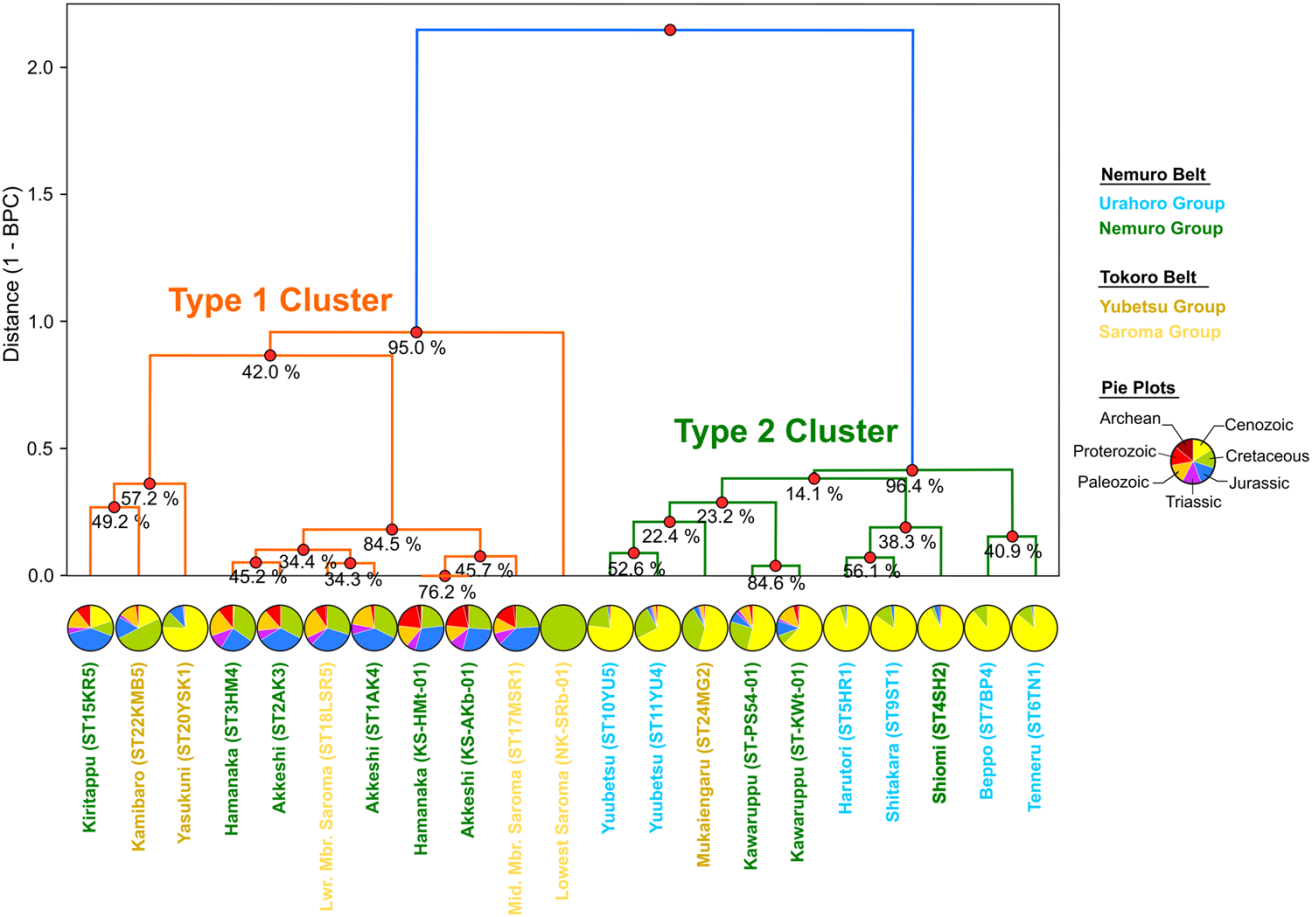
Dendrogram exhibiting the results of cluster analysis of the detrital zircon U–Pb age distributions obtained in the Paleo-Kuril Arc. The pie charts show the frequency of zircon ages for each age category. The percentages below the cluster nodes (red circles) indicate the probability that each cluster is supported in the 1000 resample analysis from the MCMC chain. See the text for method details. Two clusters (Type 1 and 2) are recognized in the dendrogram with > 95% confidence: all samples in the Type 1 cluster have YSG ages before 59 Ma, whereas all samples in the Type 2 cluster have YSG ages after 52 Ma.

The cluster analysis based on the Bayesian Population Correlations showed that the age distributions of the samples from the Paleo-Kuril Arc is classified into two clusters: Types 1 and 2 (Fig. 3). In addition to the samples of this study, data from previous literature^6,7^ were included in this analysis. The samples from the Lower and Middle Saroma Group (ST18LSR5 and ST17MSR1), the Hamanaka (ST3HM4 and KS-HMt-01), the Akkeshi (ST1AK4, ST2AK3, and KS-AKb-01), and the Kiritappu (ST15KR5) Formations of the Nemuro Group, and the Kamibaro (ST22KMB5) and the Yasukuni (ST20YSK1) Units of the Yubetsu Group were classified into Type 1. This cluster was supported with high confidence (95.0%). Inside this Type 1 cluster, the samples from the Nemuro Group (the Akkeshi and Hamanaka Formations) and the Saroma Group (Lower and Middle Groups) comprises a cluster with a confidence of 84.5%. Another minor cluster with 57.2% confidence was composed of the samples from the Kiritappu Formation of the Nemuro Group and the younger units of the Yubetsu Group (the Kamibaro and Yasukuni Units).

The sample of the Lowest Saroma Group measured by Nanayama et al.^7^ indicated intermediate Bayesian Population Correlations values (0.06–0.58) with Type 1 distributions (Fig. 3). In contrast, it exhibited very low values (0.03–0.52) with Type 2 distributions; thus, it was also classified as a Type 1 cluster.

Meanwhile, the second cluster Type 2 included the age distributions of the Kawaruppu (ST-KWt-01 and ST-PS54-01) and the Shiomi (ST4SH2) Formations of the Nemuro Group, all formations of the Urahoro Group, and the Mukaiengaru (ST24MG2) Unit of the Yubetsu Group. The confidence of this cluster was very high (96.4%), and the age distributions in each group exhibited very high Bayesian Population Correlations values around or above 0.8, which indicated that the features of those distributions were quite similar (Fig. 3). The samples from the Kawaruppu Formation (ST-KWt-01 and ST-PS54-01) and the Mukaiengaru (ST24MG2) Unit exhibited very high Bayesian Population Correlations values (0.66–1.00) with Type 2 samples but also moderate values (0.40–0.80) with Type 1 samples.

## Discussion

### Origin of the Paleo-Kuril Arc

The origin of the Paleo-Kuril Arc has been still debated. Recently, two different hypotheses have been developed for the tectonic history of the Paleo-Kuril Arc. The first is that the arc originated as an intraoceanic arc^2,5,9^, and the second hypothesis is that the arc originated as a continental arc and experienced ridge subduction^3,6,11,34–36^.

The first hypothesis infers that the inversion of the mid-oceanic ridge between the Izanagi and Pacific Plates produced the subduction zone before 80 Ma (e.g., Domeier et al.^2^). If this reconstruction of the history of the Izanagi Plate, the older continental crusts including micro-continental blocks unlikely existed along this subduction zone because the boundary was initially the ridge. Thus, if the Paleo-Kuril Arc system developed along the boundary between the Izanagi-Pacific Plates as the intraoceanic arc, the sediment in this arc system should not contain the Precambrian zircon grains abundantly, even if any, the presence should be only a minimal number of reworked particles.

The sample of sedimentary rocks belonging Type 1 Cluster of the Paleo-Kuril Arc contained a large number of zircon grains from Precambrian to Paleozoic ages with syn-sedimentary grains (i.e., ~ 70 Ma). Thus, the deposits of the Paleo-Kuril Arc are the mixtures of grains from the active plate margin and the continental cratons. Notably, samples of the Campanian Lower Saroma Group in the Tokoro Belt, the oldest forearc deposit in the Paleo-Kuril Arc, were classified into Type 1 cluster containing Precambrian zircons. These zircon grains indicated that the Paleo-Kuril Arc was connected with the continental crust at the beginning of the arc system.

The Precambrian detrital zircon grains included in the Type 1 Cluster are very unlikely to be the inherited grains transported far from the intraoceanic island arc. Volcanic ash containing minor inherited zircon grains from continental margins can be transported by winds across the plate boundary, and turbidity currents may scour them to transport the forearc basin. In that case, the inherited zircons must be included as very minor components. However, the Nemuro Group samples (Type 1) contain significant amounts of Precambrian zircons (Fig. 2), which often exceeds 20% in all detrital zircon grains. It would be difficult to interpret that they were not directly supplied from the continental cratons. Indeed, the forearc deposits of the Izu-Bonin Arc, which is the intraoceanic arc between the Pacific and the Philippine Sea Plates, only contain detrital zircons exhibiting syn-depositional ages without any Precambrian zircons, although they are close to the Eurasian Continental Plate^37^. Another example is the Talkeetna arc^38^ in Alaska, which was originally developed as the oceanic arc. The Jurassic forearc deposits of the arc contain no Precambrian zircon, while the Cretaceous deposits contain minor (< 0.7%) Precambrian grains because the arc collided with the continent at that time. In contrast, Precambrian zircons are abundant in sandstones deposited before the Late Cretaceous in the SW and NE Japan Arc systems that were continental arc systems linked with the North China Craton (e.g., Isozaki et al.^37^).

Therefore, this study supports the second hypothesis of the tectonic evolution of the Paleo-Kuril Arc based on U–Pb geochronological data obtained from sandstone samples in the Nemuro and Tokoro Belts. Type 1 detrital zircon age distributions containing Precambrian zircon ages in the Tokoro and Nemuro Belts samples implied that the Paleo-Kuril Arc’s origin was a continental magmatic arc, not an intraoceanic arc system isolated from the continental sediment provenance.

The most probable sediment source of the Paleo-Kuril Arc is the Okhotsk Block's continental crust that lies beneath the Sea of the Okhotsk, or the Siberian Craton behind it. Samples obtained by dredging in the central region of the Sea of Okhotsk indicate that the Okhotsk Block contains many Cretaceous igneous rocks of magmatic arc origin^39^. Its basement has been considered as a continental crust^40–42^ or a complex accretionary structure of the Eurasian Plate after the oceanic crust stopped subducting. The age of the Okhotsk Sea basement is estimated ~ 65 Ma (Cretaceous–Paleocene boundary)^41^ and older (~ 200 Ma)^40^. The basement consists of metabasaltic, metadioritic, granite-metamorphic and sedimentary-volcanogenic layers with total thickness around 25–30 km^40^. The structures of basement compose of crustal faults, rifts, folds, and subduction zones^42^. The igneous rocks collected by dredging were dated as 85–95 Ma (Late Cretaceous), which is slightly older but essentially similar to the depositional age of the Lower Saroma Group. It has yet to be clarified whether the volcanic arc forming the central part of the Okhotsk block was a continental or intra-oceanic arc. However, new study of petrological and geochemical analyses from volcanic rocks in the Okhotsk Sea suggested that the Okhotsk Block was a continental magmatic system^43^. That is consistent with study from Lelikov et al.^44^ that analyzed of Late Cretaceous granitoids and volcanic rocks from dredged samples in the central of the Okhotsk Sea, suggesting the continental setting developed in the basement of the Okhotsk Sea (Okhotsk Block). Bindeman et al.^45^ analyzed the sample from the Sreddiny Massif in the Kamchatka Peninsula and yielded a broad spectrum of zircon age distributions, ranging from Precambrian to the Late Cretaceous. They suggested that the Sreddiny Massif was a part of the Okhotsk Block with sediment sourced from the Siberian continent and local continental magmatic arc. Harisma et al.^6^ identified the similar pattern of detrital zircon age distribution between samples from the Sreddiny Massif and the Nemuro Belt, suggesting the Paleo-Kuril Arc also developed around the margin of the Okhotsk Block. Thus, the Paleo-Kuril Arc may have been part of those arc systems.

The similarity of the age distribution patterns (Type 1) in the Tokoro and Nemuro Belts indicated that the sediment in both tectonic belts was derived from the same source (Fig. 2). Very high values (close to unity) of the Bayesian Population Correlations implied that the Saroma Group and the formations in the Nemuro Group shared a provenance during their deposition from the Late Cretaceous to the Early Eocene (Fig. 3). A previous study suggested that detrital zircon grains obtained from the Late Cretaceous metamorphic rocks in the Sredinny Massif of the Kamchatka Peninsula exhibited a similar age distribution to Type 1 sandstones in the Nemuro Belt^6^. Thus, the sediment provenance of the Tokoro Belt was estimated to be similar to those of other tectonic belts around the Okhotsk Block. The paleocurrent direction of turbidites in the Tokoro Belt was from southeast or northeast^46^. Considering the nearly 90° clockwise rotation of this tectonic belt in the Paleogene^18^, these paleocurrent directions should be interpreted as the existence of the northern sediment provenance. The paleocurrent directions observed in the Nemuro Group were from the north to the south^47^. These paleocurrent directions also support the interpretation that these tectonic belts shared the same sediment provenance in the north. This line of evidence implies that the continental crust of the Okhotsk Block, now submerged but confirmed to exist beneath the seafloor, would be the most plausible source of sediments.

The Eurasian continent (e.g., the North China and South China Cratons) is also potential to become clastic sources in the Paleo-Kuril Arc region^6,37^. For example, the Precambrian basement rocks of the North China Craton show a similar age distribution to the Precambrian zircon age peaks in the detrital zircons of the Paleo-Kuril Arc. However, it is geographically unlikely that the North China Craton was a source of clastic material for the Paleo-Kuril Arc during the Cretaceous, before the collision between the Paleo-Kuril Arc and the Eurasian Plate.

Nanayama et al.^7^ suggested that the Paleo-Kuril Arc was initially an intraoceanic arc because their sample from the Lowest Saroma Group did not contain Precambrian zircons. However, they analyzed the concordant U–Pb ages of 26 grains from this formation. This study examined 84 and 104 grains from the Lower and Middle Saroma Group and detected 8 and 18 Precambrian grains, respectively. Thus, a sufficient number of grains was needed to interpret the sediment source. Yutani et al.^8^ also argued that the Nokkamappu Formation of the Nemuro Group in the Paleo-Kuril Arc was deposited in an intraoceanic juvenile arc according to the geochemistry of the igneous rocks of the group. Unfortunately, no zircon grains were recovered from the samples of the Nokkamappu Formation in this study. However, Precambrian zircons were detected in the Saroma Group, which was judged to be of the same age as the Nokkamappu Formation using biostratigraphy and radiometric ages. Thus, their data interpretation also needs to be reconsidered.

### Timing of provenance transition

This study identified that the provenance transition in both belts (Nemuro and Tokoro Belts) of the Paleo-Kuril Arc occurred simultaneously. This study recognized two clusters of age distribution patterns of detrital zircons as described above, with samples from the Yasukuni, Kamibaro, and Kawaruppu formations exhibiting characteristics between them (Figs. 2, 3). These samples share a strong peak in syn-sedimentary ages and contain very few Mesozoic and Paleozoic zircon grains. Although the age distributions of these samples resemble each other, the most prominent peaks in the samples of the Kawaruppu Formation are slightly younger than others. They are, therefore, classified as a Type 2 cluster. The Kamibaro Formation sample, on the other hand, contains a relatively high proportion of Mesozoic zircon grains and is, therefore, probably assigned to the Type 1 cluster. In contrast, the Yasukuni Formation sample is intermediate in age distribution and could be assigned to either type. However, the prominent peak of this sample is slightly older than those of the Kawaruppu Formation, and thus, the Type 1 cluster is probably assigned to the Yasukuni Formation. Since the Bootstrap confidence values are all low (Fig. 3), the position of these samples within the cluster is tentative, and the cluster analysis results may change as more data are collected. Nevertheless, it is noteworthy that the sandstone beds, which have intermediate age distributions of detrital zircons, are dated to about 63–52 Ma in depositional age. We infer that this period is when a change in the source of clastic materials in this area occurred.

The detrital zircon grains in Type 1 sandstones were a mixture of syn-sedimentary volcanic origin and older grains sourced from continental cratons, whereas the detrital zircon grains in Type 2 sandstones were possibly derived only from in-situ volcanic regions. In the Nemuro Belt, a transition of the sediment provenances occurred during the Paleogene between the Kiritappu and Shiomi Formations (Fig. 2). The youngest graphical ages peak (YPP age) of these Formations were 62 Ma (early Paleocene) and 54 Ma (early Eocene), respectively. The Kawaruppu Formation exhibiting Type 2 age distribution was estimated to have been deposited in ~ 52 Ma, supporting the provenance transition occurred by the Early Eocene. In the Tokoro Belt, a transition also occurred during the Paleogene between the Yasukuni (62 Ma) and the Mukaiengaru (54 Ma) Units. Thus, we postulated that the timing of the provenance transition occurred simultaneously in the Nemuro and Tokoro Belts of the Paleo-Kuril Arc in the Early Eocene.

It is too early to conclude the cause of the sediment provenance transition in the Paleo-Kuril Arc since there are many possible causes. However, such a widespread and simultaneous change in the source of sediments is generally related to a significant tectonic event. This opening of the back-arc basin (Kuril Basin) could be a cause of the provenance transition, but it was estimated to have occurred in the Miocene^48,49^. The new radiometric age study of Werner et al.^50^ revealed the time of the opening of the Kuril Basin was expected at the late Oligocene (25.3–25.9 Ma). Both ages show younger, which is unlikely to be a cause of the provenance transition in the Paleo-Kuril Arc. The timing of this sediment provenance transition in the Paleo-Kuril Arc was slightly before the period when the Izanagi-Pacific ridge subduction was estimated to have occurred^6^. Kimura et al.^3^ suggested that ridge subduction occurred in the early Paleogene around the western Pacific region as described above. For example, it has been suggested that the ridge subduction ceases igneous activity, but the subduction of young oceanic plates before and after the ridge subduction increases the volcanic activity. If a sizeable volcanic range were to develop on the volcanic front of the Kuril Arc, which is currently developing on the north side of the tectonic belt originating in the Paleo-Kuril Arc, in conjunction with volcanic activity immediately after the ridge subduction, it would have become a barrier to the supply of debris from the northern continental crust. The oblique collision of the Paleo-Kuril Arc with the Tohoku Arc also caused massive flexural deformation of the crust, which may also have caused the formation of the mountain range. However, the development of such a mountain range is only a hypothesis at this point. The influence of ridge subduction into the Paleo-Kuril Arc must be explored in future studies.

## Material and methods

In this study, we newly measured U–Pb ages of 537 detrital zircon grains from one sandstone sample from the Nemuro Belt (Fig. 1b) and five sandstone samples from the Tokoro Belt (Fig. 1c) using laser ablation–inductively coupled plasma–mass spectrometry at the University of Tokyo (see the methodological details in the supplementary material). Detrital zircon grains were separated using the standard mineral separation techniques at Kyoto University. The first process was rock fragmentation, where sandstone samples were crushed using a mortar rock crusher and were sieved with 246 µm mesh. The second process was micro-separation, which involved panning, magnetic separation to separate grains from magnetic minerals, and gravity separation using a heavy Sodium Polytungstate (NaPT) solution. About 200 zircon grains from sandstone sample were mounted under a binocular microscope, embedded in a 10 mm epoxy resin disk, and continued to polish process to approach half of the zircon grains thickness. We mounted zircons in different grain sizes and shapes to obtain age information in this study. The zircon imaging process was conducted through two steps. The first step was to photograph zircon grains under transmitted light and reflected light using an optical microscope. The second step was to take images of cathodoluminescence (CL) and backscattered electron (BSE) using JEOL Superprobe JXA-8105. To avoid contamination during sample preparation, we conduct these following instructions: washing the rock sample using running water and continue to dry step before it is crushed; cleaning the equipment using running water and air compressor during sample preparation steps (e.g., crushing, sieving, and panning) to ensure the contamination risks are low; and cleaning the microscope and surrounding equipment using ethanol to ensure those clear by foreign particles from previous mount. In the Tokoro Belt (Fig. 1c), we examined two samples from the Saroma Group (ST17MSR1 and ST18LSR5) and three samples from the Yubetsu Group (ST20YSK1, ST22KMB5, and ST24MG2) (Table 1). In the Nemuro Belt (Fig. 1b), one sample was taken from the Kiritappu Formation (ST4KR5) (Table 1).

**Table 1:** Summary of detrital zircon analyses of sandstone samples in the Nemuro and Tokoro Belts.

Formation/unit	Coordinates	No. of U–Pb analyses	Biostratigraphy/radiometric age	MDA	
				YSG age (Ma ± 2σ)	YPP age (Ma)
Urahoro Group (Nemuro Belt)					
Shitakara (ST9ST1)	E 144° 29′ 33.61″ N 42° 56′ 48.81″	85 (104)^(a)^	Late/Middle Eocene^(a)^	42.9 ± 1.3^(a)^	49^(a)^
Yuubetsu (ST10YU5)	E 144° 27′ 03.13″ N 42° 56′ 42.22″	82 (104)^(a)^	Middle Oligocene^(a)^	42.1 ± 2.1^(a)^	48^(a)^
Yuubetsu (ST11YU4)	E 144° 03′ 48.06″ N 43° 12′ 59.91″	74 (104)^(a)^	Middle Oligocene^(a)^	45.1 ± 1.6^(a)^	47^(a)^
Tenneru (ST6TN1)	E 144° 28′ 37.99″ N 42° 56′ 34.33″	86 (104)^(a)^	39.1 ± 0.2 Ma^(a)^	37.2 ± 1.1^(a)^	39^(a)^
Harutori (ST5HR1)	E 144° 28′ 00.01″ N 42° 56′ 41.57″	76 (104) ^(a)^	39.5 ± 0.2 Ma and 40.8 ± 1.1 Ma^(a)^	38.7 ± 1.2^(a)^	55^(a)^
Beppo (ST7BP4)	E 144° 27′ 51.95″ N 42° 56′ 55.36″	73 (104) ^(a)^	N/A (no fossil recorded)^(a)^	39.8 ± 1.2^(a)^	40^(a)^
Nemuro Group (Nemuro Belt)					
Shiomi (ST4SH2)	E 144° 28′ 19.16″ N 42° 56′ 49.92″	76 (104)^(a)^	Selandian^(a)^	52.1 ± 0.8^(a)^	54^(a)^
Kiritappu (ST15KR5)	E 145° 08′ 26.96″ N 43° 04′ 51.94″	65 (65)	Danian	59.0 ± 1.5	62
Akkeshi (ST1AK4)	E 145° 07′ 41.56″ N 43° 08′ 07.40″	68 (104)^(a)^	Danian^(a)^	69.6 ± 2.0^(a)^	70^(a)^
Akkeshi (ST2AK3)	E 145° 08′ 14.28″ N 43° 08′ 24.32″	86 (104)^(a)^	Danian^(a)^	71.5 ± 1.6^(a)^	72^(a)^
Akkeshi (KS-AKb-01)	E 145° 13′ 45.05″ N 43° 08′ 52.71″	103 (116)^(b)^	Maastrichtian–Danian^(b)^	66.8 ± 1.7^(b)^	
Hamanaka (ST3HM4)	E 145° 13′ 09.08″ N 43° 08′ 20.47″	92 (117)^(a)^	Lower Maastrichtian^(a)^	65.8 ± 3.6^(a)^	71^(a)^
Hamanaka (KS-HMt-01)	E 145° 14′ 19.01″ N 43° 09′ 9.83″	116 (116)^(b)^	Maastrichtian^(b)^	65.6 ± 4.3^(b)^	
Kawaruppu (ST-PS54-01)	E 143° 48′ 9.46″ N 43° 06′ 13.31″	39 (41)^(b)^	Early Selandian–early Lutetian^(b)^	52.7 ± 7.0^(b)^	
Kawaruppu (ST-KWt-01)	E 143° 48′ 55.77″ N 43° 05′ 32.11″	111 (120)^(b)^	early Selandian–early Lutetian^(b)^	52.9 ± 1.3^(b)^	
Saroma Group (Tokoro Belt)					
Middle Mbr. (ST17MSR1)	E 143° 54′ 46.75″ N 44° 04′ 08.34″	104 (104)	Coniacian–Santonian	71.9 ± 3.9	74
Lower Mbr. (ST18LSR5)	E 143° 54′ 35.12″ N 44° 04′ 11.88″	84 (88)	Coniacian–Santonian	69.2 ± 2.3	70
Lowest Unit (NK-SRb-01)	E 143° 52′ 58.29″ N 44^o^ 00′ 25.07″	26 (32)^(b)^	Maastrichtian^(b)^	76.9 ± 3.1^(b)^	
Yubetsu Group (Tokoro Belt)					
Yasukuni (ST20YSK1)	E 143° 35′ 48.49″ N 44° 03′ 57.82″	94 (100)	*Undetermined*	61.2 ± 2.2	62
Kamibaro (ST22KMB5)	E 143° 39′ 22.84″ N 44° 06′ 43.69″	89 (91)	*Undetermined*	63.1 ± 1.9	64
Mukaiengaru (ST24MG2)	E 143° 32′ 27.54″ N 44° 05′ 37.43″	101 (104)	Early Eocene	52.5 ± 1.2	54

*MDA* maximum depositional age, *Mbr* member, *YPP* the youngest graphical age peak, *YSG* the youngest single-grain age.

^(a)^Harisma et al.^6^; ^(b)^Nanayama et al.^7^.

We also compiled data from previous studies in the Paleo-Kuril Arc that contained the U–Pb ages of 1210 grains from 16 formations^6,7^. They exhibited multimodal or unimodal age distributions depending on the horizons (Fig. 2)^6,7^. A previous study by Nanayama et al.^7^ identified five sandstone samples from the Nemuro and Tokoro Belts with a range of zircon grains from 32 to 116 grains: two samples from the Kawaruppu Formation (ST-PS54-01 and ST-KWt-01), one sample from the Lowest Saroma Group (NK-SRb-01), and two samples from the Akkeshi (KS-AKb-01) and the Hamanaka (KS-HMt-01) Formations of the Nemuro Group were analyzed in this study (Table 1). We also analyzed ten sandstone samples measured by Harisma et al.^6^: six samples were from the Urahoro Group (ST9ST1, ST10YU5, ST11YU4, ST6TN1, ST5HR1, and ST7BP4) and four samples were from the Nemuro Group (ST4SH2, ST1AK4, ST2AK3, and ST3HM4) (Table 1).

The age distributions of measured samples described above were then reconstructed by the Bayesian estimation, and the Bayesian Population Correlations were calculated to examine the similarity of the age distributions of detrital zircon grains^51^.

This study performed a hierarchical cluster analysis to understand the relationship between each age distribution. As mentioned above, the similarity between two age distributions can be measured by Bayesian Population Correlations. In existing studies, it has often been debated how many particles should be measured when comparing age distributions of detrital zircon grains. Although there are claims that 117 particles or 60 particles are a sufficient number^52,53^, however, both of these are thresholds that do not take into account the characteristics of the original zircon age distribution. Previous studies have pointed out that, depending on the distribution's characteristics, measuring more than 300 particles may be necessary to capture all minor peaks of age distributions^51^. Such large number of grain measurements for every sample is too time-consuming to evaluate tectonic history of broad regions. If samples less than 300 particles are not used, almost all of the data from existing studies cannot be referenced, resulting in a significant loss of information.

To resolve this situation, it is necessary to conduct an analysis that considers the data's uncertainty rather than always requiring a reliable estimation of the population distribution. The Bayesian Population Correlation (BPC) was developed as an index to compare the characteristics of clastic zircon age distributions, taking into account the estimation uncertainty^51^. The Bayesian Population Correlation is a measure that reveals differences between two zircon age distributions by comparing two target scenarios based on likelihood. The first scenario is that the two samples of clastic zircons came from precisely the same age population, while the second scenario is that they came from entirely different age populations. For calculating this index, age distributions of detrital zircons for each sample are used to perform a Bayesian inference for the age distribution that produced it. Next, the likelihood is calculated according to the two scenarios described above, and the Bayesian Population Correlation is obtained as the ratio of the two likelihoods, corrected for the effect of the number of grains. The larger the value, the more likely it is that the two zircon age distribution data came from the same age population. On the other hand, if two sandstone samples were sourced from rocks with entirely different zircon age distributions, the Bayesian Population Correlation becomes 0. If the number of particles measured in the sample is small or if minor peaks in the age distribution are important comparative indicators, the width of the Bayesian confidence intervals in the Bayesian Population Correlation becomes large, allowing us to quantitatively discuss the confidence level of the similarity of the age distributions. Numerical experiments have also verified that the Bayesian Population Correlation estimates show almost no bias, even when the number of particles is small. Further details are given in the paper proposing this index^51^.

We performed 1000 times resampling from the chains of the Markov Chain Monte Carlo method (MCMC) for estimating Bayesian Population Correlations. We subtracted the mean of the Bayesian Population Correlations in the sample from 1 to obtain the distance between the two age distributions. From the distance matrix between the age distributions, Ward's method constructed a dendrogram of hierarchical clusters. Then, cluster analysis was performed on each of the resampled age distributions, and the diversity of the results was used to evaluate the uncertainty of the cluster analysis. The resulting of dendrogram exhibits the percentage probability of having the same composition of the clusters in the resampled age distributions.

In this study, we identified the maximum depositional ages (MDA) of sedimentary rock samples from measured sections of the Nemuro and the Tokoro Belts complex that were estimated from the ages of the youngest single grain (YSG) and the youngest graphical peak (YPP) based on the method of Dickinson and Gehrel^54^. The youngest single grain is measured from the age of the youngest single grain, while the youngest graphical peak is obtained by identifying the first age peak from the age distribution of detrital zircon grains^54^. The youngest single grain could be unreliable due to the diagenetic process or measurement problems such as Pb loss, so that the stratigraphic context and the discrepancy between the values of the youngest single grain and the youngest graphical peak were considered for judging its reliability. The existing biostratigraphic age estimations were also referred to examine the reliability of the MDAs estimated by detrital zircons.

### Supplementary Information

Supplementary Information 1.

Supplementary Information 2.

## Data Availability

All data generated and compiled in this study are presented in the Supplemental Information. The data and codes for cluster analysis by comparing clastic zircon age distributions are provided in Zenodo at https://doi.org/10.5281/zenodo.10629549.
