# Supplementary material for: Cretaceous to early Paleogene sediment provenance transition from continental to magmatic arc systems in the Northwestern Pacific Region
Source: Sci Rep. 2024 Mar 27;14:7280. doi: 10.1038/s41598-024-55471-1 (PMC10973497; doi:10.1038/s41598-024-55471-1)
Supplement: Supplementary file 1 — Supplementary Information 1. [file 41598_2024_55471_MOESM1_ESM.docx]

**Supplemental Materials**

**Figure S1.** Photos of the selected outcrops of the study area.

**Figure S2.** CL and BSE images of selected detrital zircons of the study area.

**Figure S3.** DZ age distributions for Type 1 and Type 2 in the Nemuro and the Tokoro Belts.

**Figure S4.** The Th/U ratio of zircon grains from the Nemuro and the Tokoro Belts.

**Figure S5.** The BPC values of the Nemuro and Tokoro Belts.

**Figure S6.** The t-SNE diagram.

**Table S1.** Instrumentation and operational setting of the LA-ICP-MS in this study.

**Table S2.** U-Pb geochronological datasets.


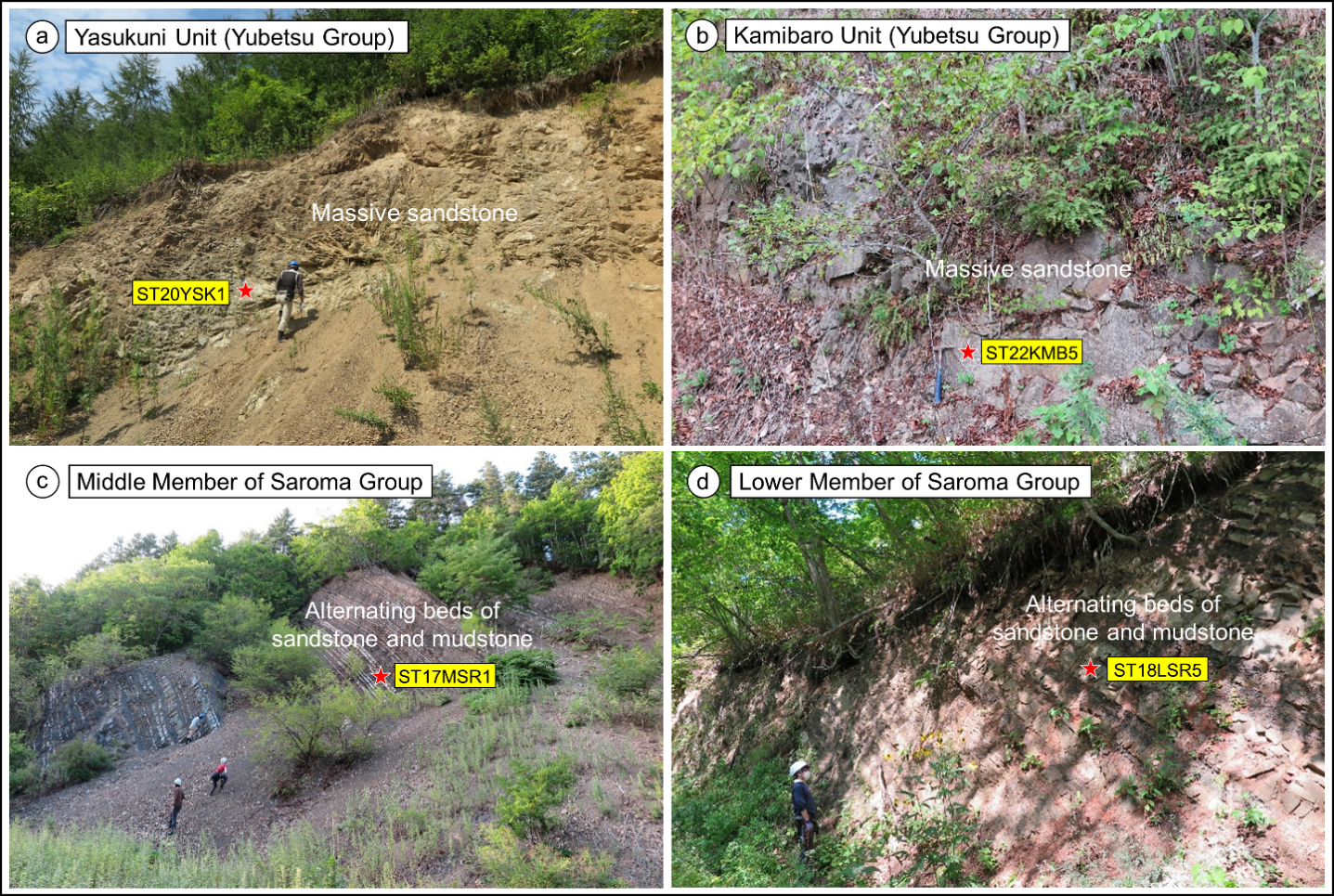


**Figure S1.** Photos of the selected outcrops of the study area. (a) Yasukuni Unit (Yubetsu Group), (b) Kamibaro Unit (Yubetsu Group), (c) Middle Member of the Saroma Group, and (d) Lower Member of the Saroma Group. The red star shows the location of the sample.


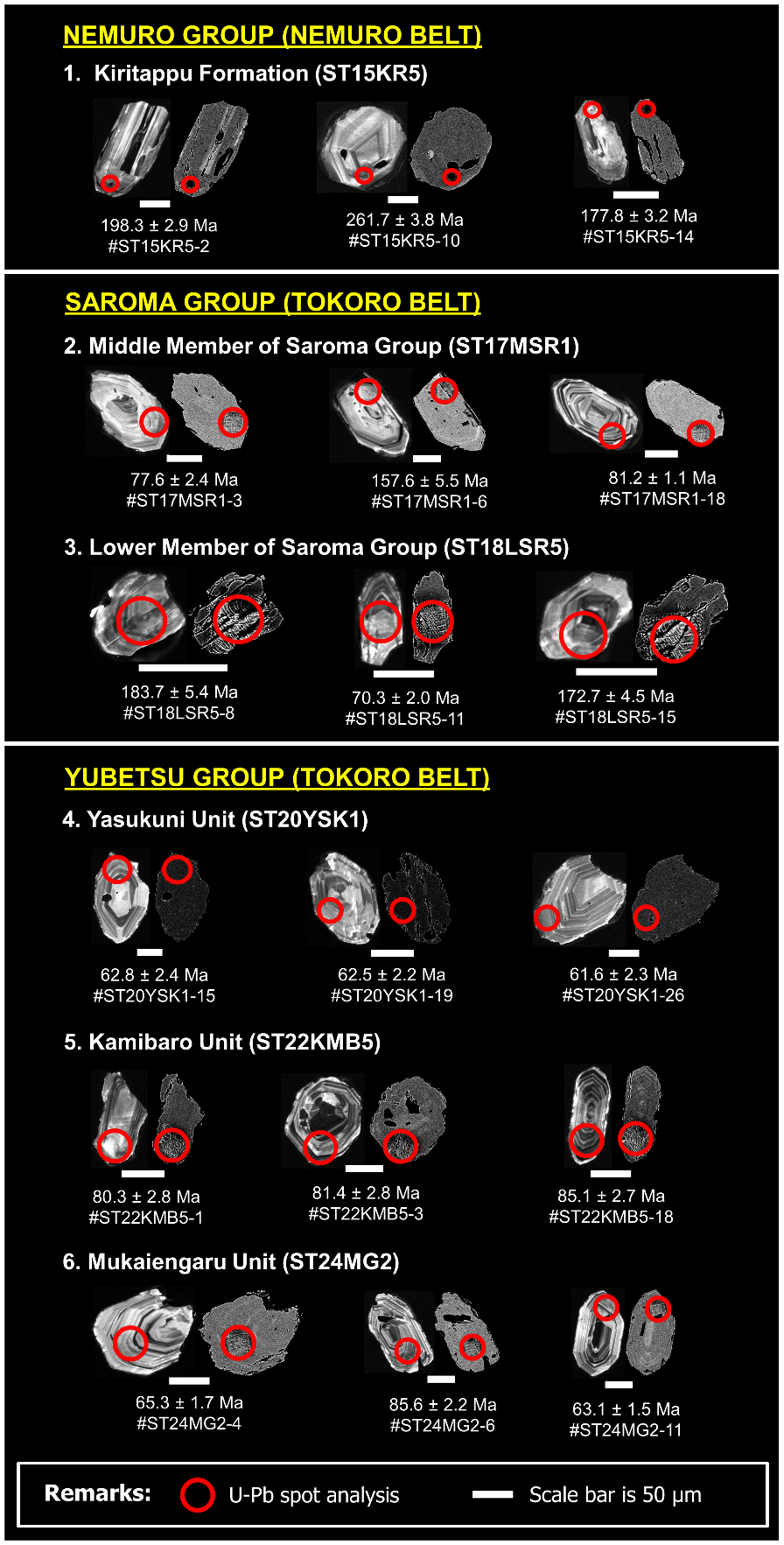


**Figure S2.** Cathodoluminescence (CL) and backscattered electron (BSE) images of selected detrital zircons of the study area. Red circles indicate the locations of the U-Pb spots. White scale bars are 50-μm long.

**
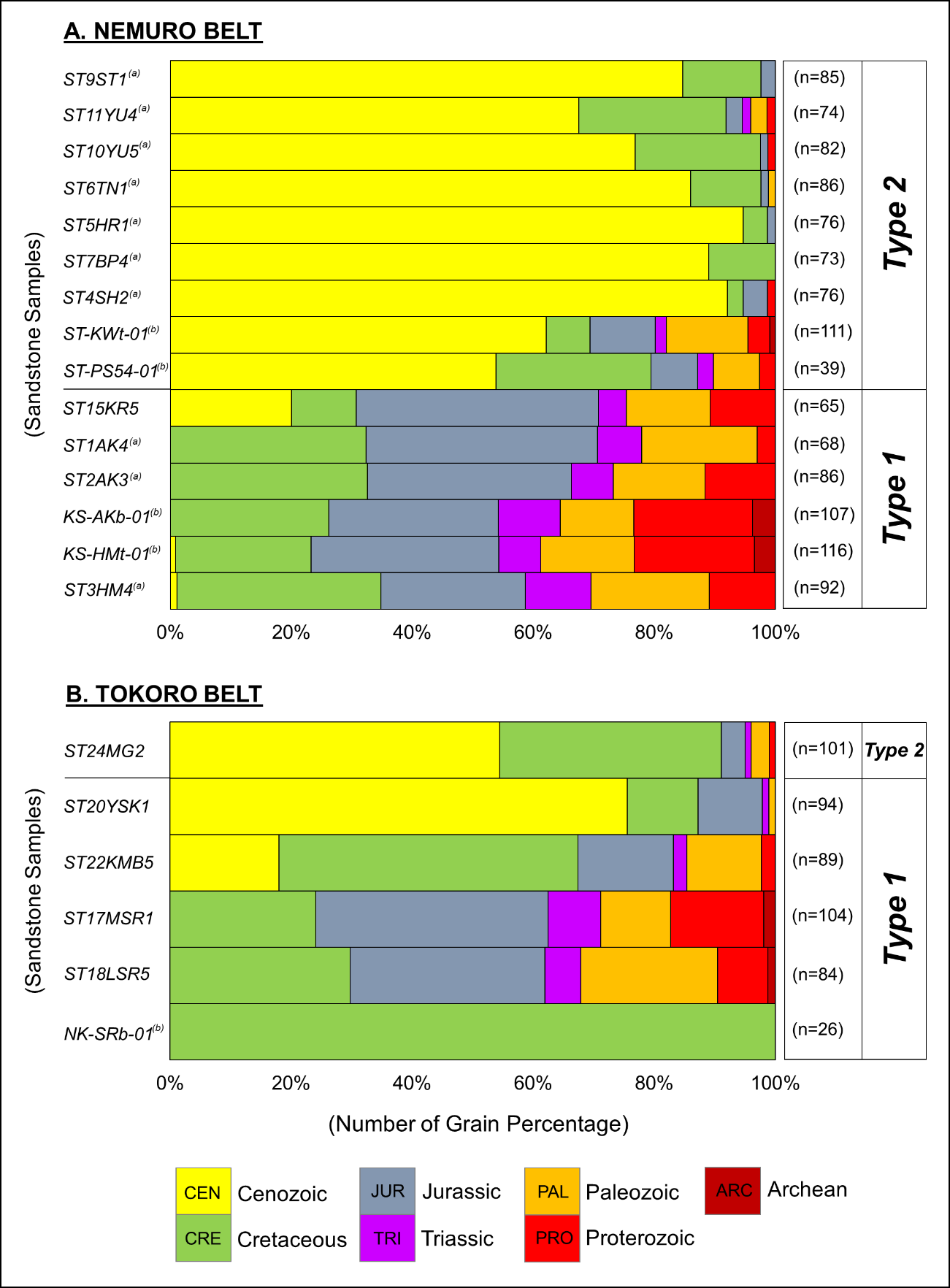
**

**Figure S3.** Stacked graph showing percentages of different components of detrital zircon age distributions for Type 1 and Type 2 sandstones in the Nemuro and the Tokoro Belts through time. References: (a)^1^ and (b)^2^.


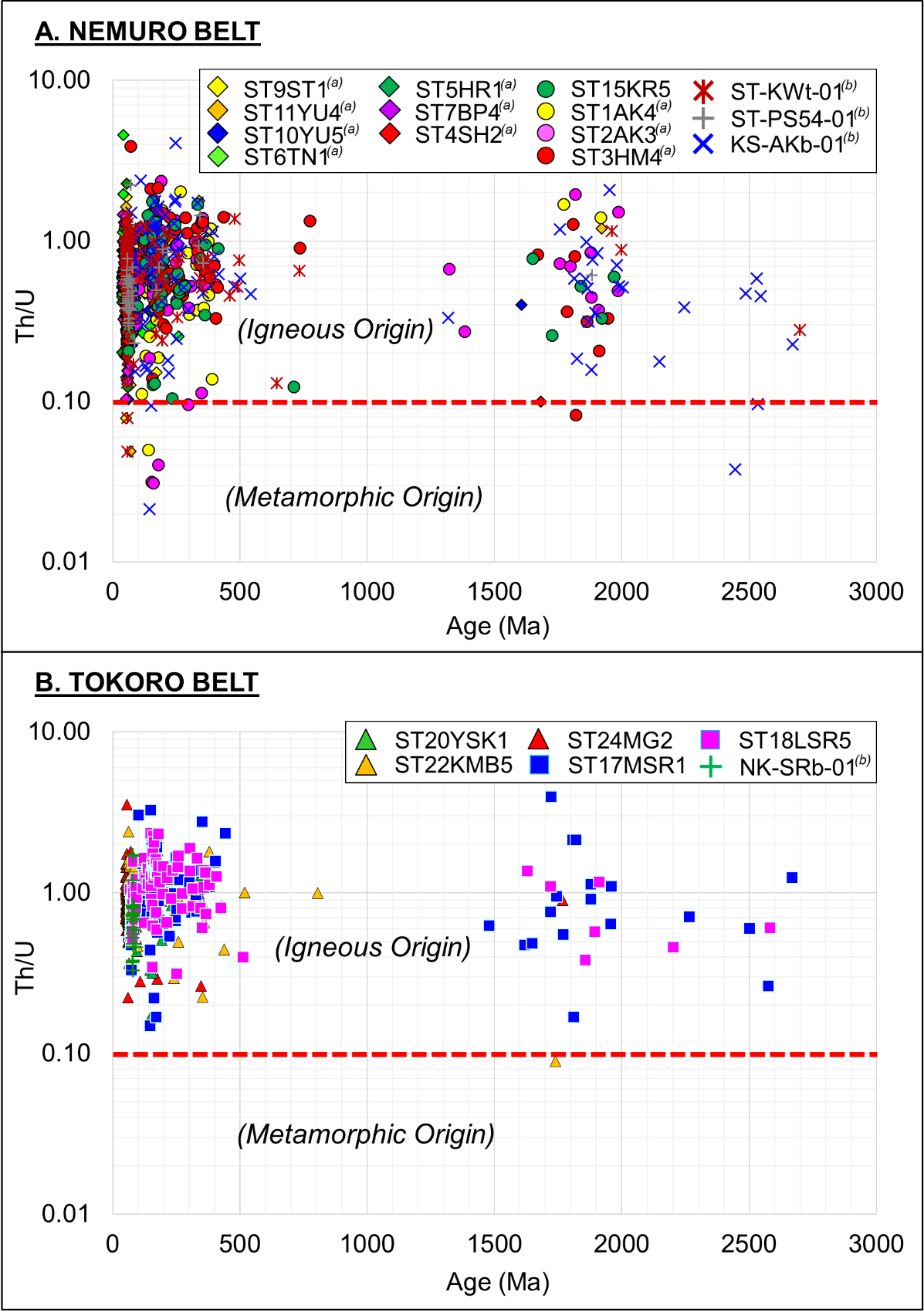


**Figure S4.** The Th/U ratio of zircon grains from (a) Nemuro Belt and (b) Tokoro Belt. The zircon grains with Th/U > 0.1 usually indicate a magmatic origin, and Th/U < 0.1 likely to demonstrate a metamorphic origin (e.g., Hoskin and Black^3^; Rubatto^4^). References: (a)^1^ and (b)^2^.


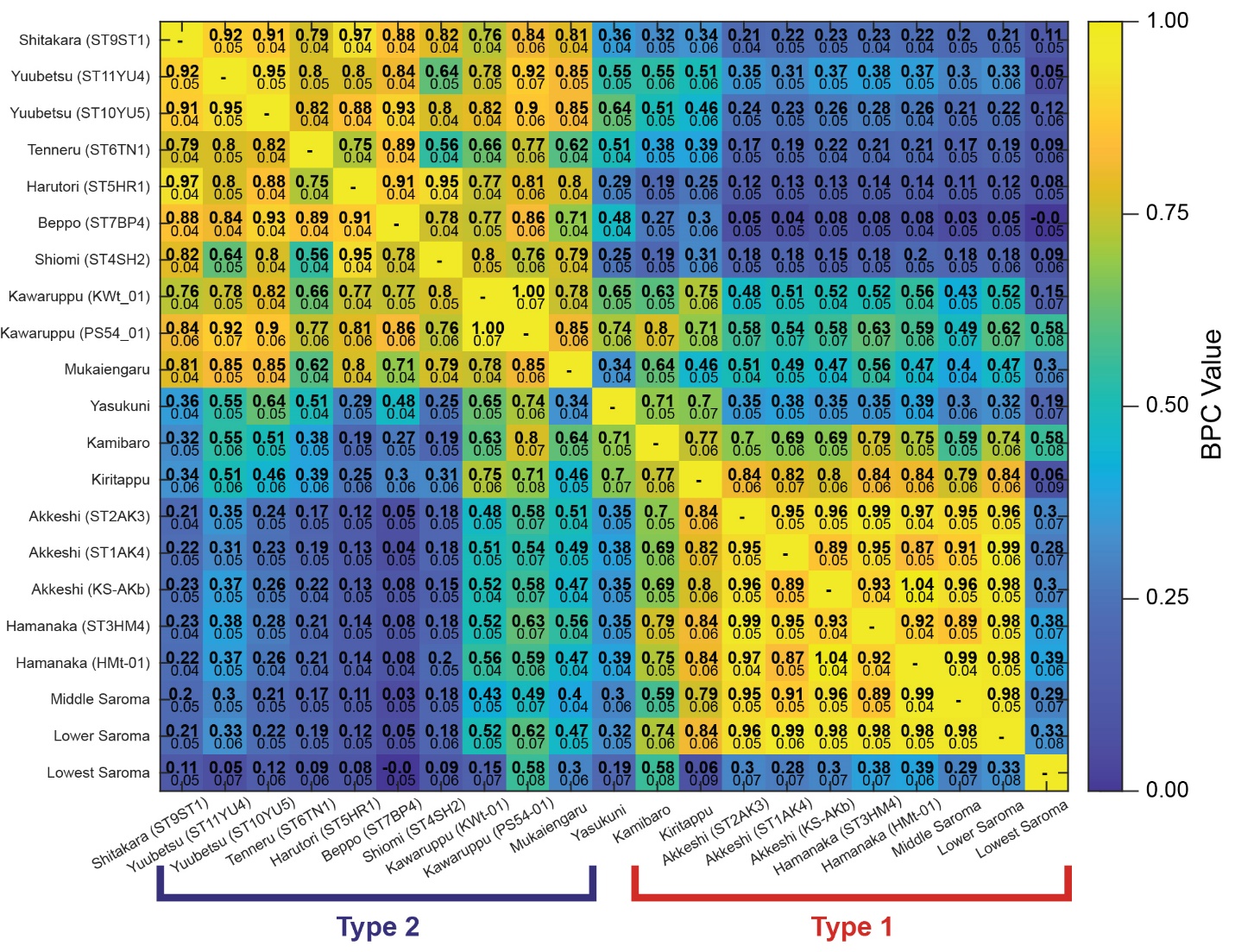


**Figure S5.** BPC values^5^ between the measured age distributions in the Nemuro and Tokoro Belts of the PKA. The age distributions obtained in this study were divided into two types: Types 1 and 2.


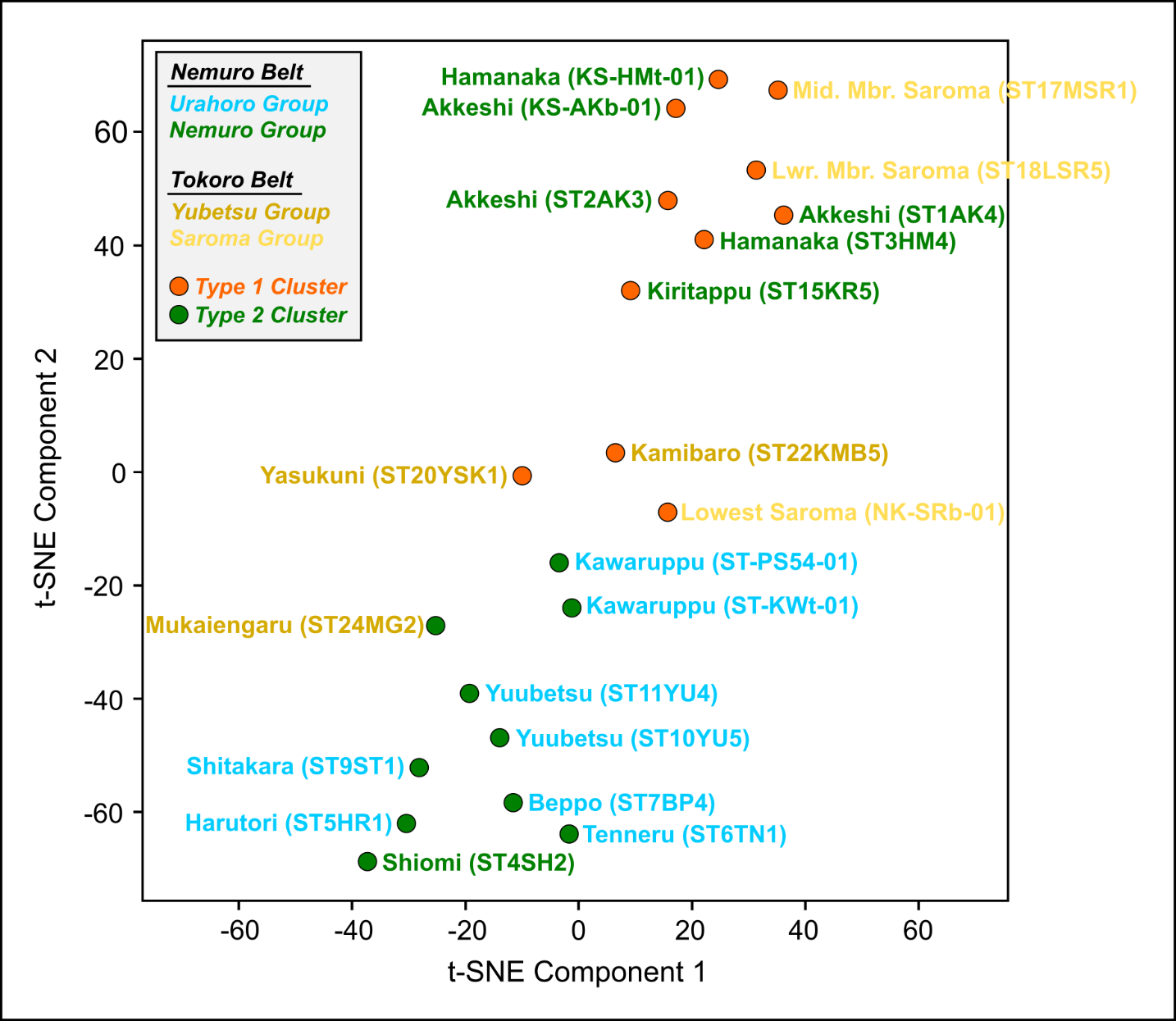


**Figure S6.** The t-SNE diagram. This diagram exhibits the Kamibaro, Yasukuni, and Kawaruppu Units have intermediate features between two clusters.

**Instrumentation and Operational Setting of the LA-ICP-MS in this Study**

Zircon U-Pb ages from all samples in the Tokoro Belt were determined using laser ablation–inductively coupled plasma–mass spectrometry (LA-ICP-MS) installed at the Geochemical Research Center at the University of Tokyo. The Nu Plasma II instrument was used in this study. Measurements were conducted using a wavelength of 260 nm, a fluence of ca. 4.0 J/cm^2^, and an ablated area of 20×20 µm^2^ by raster scanning of laser spots with the spot size of 10 µm. NIST SRM 612 and Nancy 91500 zircon were used as primary references, and OD-3 and GJ-1 were used as secondary references. The U-Pb concordia diagrams and probability density plots were illustrated using IsoplotR software^6^. The information of the operational setting of the LA-ICP-MS is summarized below.

**Table S1.** Instrumentation and operational setting of the LA-ICP-MS in this study.

| **Laser Ablation System** | |
| --- | --- |
| Instrument | : Jupiter solid nebulizer |
| Laser wavelength | : 260 nm |
| Sampling mode | : Raster mode |
| Fluence | : ca. 4 J cm^-2^ |
| Spot size | : 10 µm |
| Repetition rate | : 1000 Hz |
| Ablation pit size | : 20 × 20 μm^2^ square |
| He gasses flow rate | : 0.60 L/min |
| Ar make-up gas flow rate | : 0.8 L/min |
| **ICP Mass Spectrometer** | |
| Instrument | : Nu Plasma II |
| Data reduction | : Pre-ablation shots for surface cleaning were conducted prior to isotopic analyses. After the pre-ablation cleaning, measurements of gas blank intensities for 4 seconds were followed by each spot analysis, and isotopic data were acquired for 4 seconds to integrate total ion counts during laser ablation for each spot analysis. |
| Monitored isotopes | : ^235^U, ^232^Th, ^208^Pb, ^207^Pb, ^206^Pb, ^204^Pb and ^204^Hg, and ^202^Hg |
| Detection mode | : Pulse-counting mode of secondary electron multipliers and photomultipliers for ^235^U, ^2208^Pb, ^207^Pb, ^206^Pb, ^204^Pb and ^204^Hg, and ^202^Hg, and analog mode of a Faraday cup for ^232^Th^7,8^ |
| Dwell time | : 0.1 s |
| The integration time per peak | : 4.0 s |
| Primary reference | : NIST SRM 612, Nancy 91500 |
| Secondary reference | : OD-3, GJ-1 |
| Data processing | External calibration of isotopic ratios based on the measurements of reference materials. |
| Normalization values | :^206^Pb/^238^U of Nancy 91500 zircon = 0.17928^9,10^, ^207^Pb/^206^Pb of NIST SRM 612 = 0.90726^11^, Th mass fraction of NIST SRM 612 = 37.23 µg g^-1^, and U mass fraction of NIST SRM 612 = 37.15 µg/g^12^ |

**References**

1. Harisma, H., Naruse, H., Asanuma, H. & Hirata, T. The Origin of the Paleo‐Kuril Arc, NE Japan: Sediment Provenance Change and Its Implications for Plate Configuration in the NW Pacific Region Since the Late Cretaceous. *Tectonics* **41**, (2022).

2. Nanayama, F. *et al.* Origin and evolution of the Paleo‐Kuril arc inferred from detrital zircon U–Pb chronology in eastern Hokkaido, NE Asia. *Isl. Arc* **31**, (2022).

3. Hoskin, P. W. O. & Black, L. P. Metamorphic zircon formation by solid-state recrystallization of protolith igneous zircon. *J. Metamorph. Geol.* **18**, 423–439 (2000).

4. Rubatto, D. Zircon trace element geochemistry: Partitioning with garnet and the link between U-Pb ages and metamorphism. *Chem. Geol.* **184**, 123–138 (2002).

5. Tye, A. R., Wolf, A. S. & Niemi, N. A. Bayesian population correlation: A probabilistic approach to inferring and comparing population distributions for detrital zircon ages. *Chem. Geol.* **518**, 67–78 (2019).

6. Vermeesch, P. IsoplotR: A free and open toolbox for geochronology. *Geosci. Front.* **9**, 1479–1493 (2018).

7. Hattori, K., Sakata, S., Tanaka, M., Orihashi, Y. & Hirata, T. U-Pb age determination for zircons using laser ablation-ICP-mass spectrometry equipped with six multiple-ion counting detectors. *J. Anal. At. Spectrom.* **32**, 88–95 (2017).

8. Obayashi, H., Tanaka, M., Hattori, K., Sakata, S. & Hirata, T. In situ 207Pb/206Pb isotope ratio measurements using two Daly detectors equipped on an ICP-mass spectrometer. *J. Anal. At. Spectrom.* **32**, 686–691 (2017).

9. Wiedenbeck, M. *et al.* Three Natural Zircon Standards for U‐Th‐Pb, Lu‐Hf, Trace Element and REE Analyses. *Geostand. Newsl.* **19**, 1–23 (1995).

10. Sakata, S. *et al.* A new approach for constraining the magnitude of initial disequilibrium in Quaternary zircons by coupled uranium and thorium decay series dating. *Quat. Geochronol.* **38**, 1–12 (2017).

11. Jochum, K. P. *et al.* GeoReM: A new geochemical database for reference materials and isotopic standards. *Geostand. Geoanalytical Res.* **29**, 333–338 (2005).

12. Pearce, N. J. G. *et al.* A compilation of new and published major and trace element data for NIST SRM 610 and NIST SRM 612 glass reference materials. *Geostand. Newsl.* **21**, 115–144 (1997).
